# Quality of life and anxiety in women with breast cancer before and after treatment

**DOI:** 10.1590/1518-8345.2258.2958

**Published:** 2017-12-21

**Authors:** Raquel Rey Villar, Salvador Pita Fernández, Carmen Cereijo Garea, Mª Teresa Seoane Pillado, Vanesa Balboa Barreiro, Cristina González Martín

**Affiliations:** 1 Doctoral student, Universidad de A Coruña, A Coruña, A Coruña, Spain. RN, Universidad de A Coruña, A Coruña, A Coruña, Spain.; 2 PhD, Full Professor, Universidad de A Coruña, A Coruña, A Coruña, Spain.; 3 PhD, Associate Professor, Universidad de A Coruña, A Coruña, A Coruña, Spain.; 4 MSc, Assitant Professor, Universidad de A Coruña, A Coruña, A Coruña, Spain.; 5 PhD, Adjunct Professor, Universidad de A Coruña, A Coruña, A Coruña, Spain.

**Keywords:** Quality of Life, Women, Breast Neoplasms, Anxiety, Nurses, Nursing Care Management

## Abstract

**Objectives:**

to determine the quality of life and anxiety in patients with breast cancer and the changes they experience after treatments.

**Method:**

prospective study. Breast cancer statistics (n=339, confidence=95%, accuracy= ± 5.32%). The quality of life questionnaires (QLQ) used were QLQ C-30 and QLQ Br23, and the State-Trait Anxiety Inventory (STAI) was used for anxiety. A multivariate analysis was performed to identify variables associated with baseline quality of life and anxiety as well as pre- and post-treatment differences. Authorization was obtained from the Ethics Committee, and informed consent was provided by all patients.

**Results:**

the baseline quality of life dimensions with the lowest score were future prospects (46.0/100) and sexual enjoyment (55.7/100). The dimensions with the highest score were body image (94.2/100) and role (93.3/100). The most disturbing symptoms were insomnia, fatigue and concern about hair loss. After treatment, the dimensions of physical function, role, body image, financial concerns and symptomatology worsened, whereas emotional function and future prospects improved. Severe anxiety presented as a state (48.6%) and as a trait (18.2%). The highest baseline state anxiety was associated with married-widowed status and anxiolytic medication. The greatest trait anxiety was associated with an inactive work situation, anxiolytic medication, breast swelling and advanced stage at diagnosis. After treatment, anxiety significantly decreased.

**Conclusions:**

After treatment, the quality of life score was positively modified, while state and trait anxiety decreased.

## Introduction

The utility of quality of life measurements in daily clinical practice is widely referenced in the literature. In a systematic review published in 2010^1^, it was shown that quality of life data influenced decision making in 30.1% of medical interventions and in 63.2% of non-biomedical interventions. Therefore, treatment plans can be altered for or be contingent upon the quality of life of the patient. 

According to the literature, almost 50% of cancer patients suffer from psychiatric disorders, and anxiety and depression are generally considered to be the most important psychopathological comorbidities. More than one-third of breast cancer patients may experience psychopathological disorders^2^. Psychological morbidity is influenced by multiple backgrounds and concomitant factors that affect psychic function and quality of life. 

Several studies have shown associations between anxiety and certain sociodemographic and psychosocial variables (age at diagnosis^3^, civil status^4^, level of education^5^, history of comorbidity^4^ or history of prior treatment for anxiety and/or depression^6^).

One study on the impact of anxiety^7^ related high levels of anxiety with the intensification of physical symptoms and an increase in the perception of the adverse effects of the treatments, all of which negatively affect the quality of life and the overall health status of the patient.

Given the close relationship between the nurse and patient, nursing professionals are in a position to make an overall assessment of patients, as they attend to not only their physiological or clinical needs but also their psychological and social needs.

Several randomized clinical trials have indicated psychological benefits following interventions performed by nurses. These include stress reduction^8^, decreased anxiety, depression and improvement in physical and emotional well-being^9^. Another study showed significant improvements in insomnia, dyspnoea and financial concerns^10^. 

This study performed by the nursing staff of our breast cancer unit aims to determine the level of quality of life and anxiety as a state and as a trait in women diagnosed with breast cancer before and after treatment. This study also attempts to determine the variables associated with baseline quality of life and anxiety.

## Method

This is an observational study in which patients were prospectively followed-up and that was performed at the Breast Cancer Unit of the University Hospital Complex of A Coruña (Spain). We collected all cases with a histopathological diagnosis of breast cancer that presented from December 2013 to February 2015. Male patients and those who underwent surgery and follow-up at another centre were excluded. Patients were identified at diagnosis through the results of the histopathological findings. They were contacted during a visit to the breast cancer unit. The nurse of the unit invited those who fulfilled the inclusion criteria to participate. Two measures of quality of life and anxiety were obtained, the first after the diagnosis and the second after the end of the chemotherapy and/or radiotherapy treatment. The authorization of the ethics committee and the informed consent of each participant were obtained (Autonomous Committee of Research Ethics of Galicia, code 2013/253).

The data were obtained through interviews with patients and through a review of their medical history. From each patient, we analysed the sociodemographic characteristics, previous comorbidities using the Charlson comorbidity index^11^, gynaecological and obstetric history, family history of cancer, clinical manifestations, stage, pathological type and type of therapeutic management. The validated questionnaires that were used to measure quality of life were the European Organisation for Research and Treatment of Cancer (EORTC) quality of life questionnaire (QLQ) C-30 and the QLQ Br23^12^ ^-^ ^13^. The QLQ C-30 consists of 30 questions, with a Likert-type response (1 to 7 for the overall health status and 1 to 4 for all other items). This questionnaire evaluates overall health status, five functional scales (physical, emotional, role, cognitive and social) and the presented symptoms.

The QLQ Br23 questionnaire consists of 23 items. It measures four functional scales (body image, future prospects, sexual function and sexual enjoyment) and symptoms (of the affected breast and arm, concern about hair loss and adverse effects of systemic therapies), with a Likert-type response (from 1 to 4). The system of measurement of both quality of life questionnaires presents a score of 0 to 100. Regarding these functional scales, the higher the score, the better the quality of life; regarding the scales for the assessment of symptoms, the higher the score, the worse the symptomatology.

The questionnaire that was used to measure anxiety as a state and as a trait was the State-Trait Anxiety Inventory (STAI)^14^. This questionnaire comprises two separate scales of 20 items each for the measurement of anxiety as a state and as a trait, with a Likert-type response (0 to 3). Scores range from 0 to 60 points, with higher scores indicating higher anxiety.

Justification of the sample size: 339 patients were included in the analysis of the general characteristics, which allows for a 95% confidence interval and an accuracy of ±5.32% in the estimation of the parameters of interest.

For the comparison between quality of life measurements before and after treatment, 181 patients were studied, whereas 169 patients were studied for anxiety. This sample size allows the determination of a detected difference of at least four points between the two measurements of anxiety (standard deviation=15) with a 95% confidence interval and a statistical power of 80%.

We performed a descriptive analysis of the variables included in the study. The quantitative variables are expressed as the mean, standard deviation and median. Qualitative variables are expressed as absolute values and percentages with the estimated 95% confidence interval. 

Student’s T test or Mann-Whitney U Test, and Student’s T test for paired samples or the Wilcoxon test were used as appropriate. Moreover, McNemar’s test was used to compare pre- and post-treatment categorical variables.

The correlation between quantitative variables was calculated using the Spearman or Pearson Rho correlation coefficient. The quality of life and anxiety scores were dichotomized with values respectively lower than and greater than the median for the implementation of multivariate logistic regression models.

SPSS 19.0 software, the R program for statistical analysis and EPIDAT 3.1 were used to calculate the confidence intervals.

## Results

During the study period, 524 cases of breast cancer were diagnosed, and after a review of the inclusion and exclusion criteria, 339 participants were included in the study. In all, 1% of the diagnosed cases were men, who were excluded from the study.

Table 1 shows the characteristics of the patients who were included in the study. The mean age was 58.9 years, with a median age of 59 years. The descriptive study of the sample shows that 19.8% of the women were university educated, 38.9% were employed, 64.9% were married or lived with a partner, and 50.1% took anxiolytic medication or an antidepressant. The prevalence of overweight was 34.9%, while 17.7% of the patients were smokers.

**Table 1:** Sociodemographic variables, comorbidities and obstetric-gynaecological history. A Coruña, Spain, 2013-2015

		**n (%)**	95% CI*	**Mean ±ST†**	**Median**
**Age (years)**				**58,9 ±12,5**	**59**
**Education level**					
	**Low**	**211 (62,2)**	**56,9-67,5**		
	**Medium**	**61 (18)**	**13,8-22,2**		
	**High**	**67 (19,8)**	**15,4-24,1**		
**Employment situation**					
	**Active**	**132 (38,9)**	**33,6-44,3**		
	**Inactive**	**32 (9,4)**	**6,2-12,7**		
	**Housewife**	**43 (12,7)**	**9,0-16,4**		
	**Retired**	**132 (38,9)**	**33,6-44,3**		
**Civil status**					
	**Married/partner**	**220 (64,9)**	**59,7-70,1**		
	**Widow**	**58 (17,1)**	**12,9-21,3**		
	**Single**	**39 (11,5)**	**8,0-15,0**		
	**Divorced/separated**	**22 (6,5)**	**3,7-9,3**		
**Housework**					
	**Without help**	**195 (57,9)**	**52,4-63,3**		
	**Shared Tasks**	**98 (29,1)**	**24,1-34,1**		
	**Other**	**44 (13,1)**	**9,3-16,8**		
**Smoking habit**					
	**Smoker**	**60 (17,7)**	**13,5-21,9**		
	**Non-smoker**	**222 (65,5)**	**60,3-70,7**		
	**Former smoker**	**57 (16,8)**	**12,7-20,9**		
**Body Mass Index Categories**					
	**Under weight**	**15 (4,5)**	**2,1-6,8**		
	**Normal**	**110 (32,8)**	**27,7-38,0**		
	**Overweight**	**117 (34,9)**	**29,7-40,2**		
	**Obese**	**93 (27,8)**	**22,8-32,7**		
**Charlson Comorbidity Index**				**2,3 ±0,7**	**2**
**Age-adjusted Charlson**				**3,8 ±1,5**	**4**
**Hypertension (Yes)**		**81(23,9)**	**19,2-28,6**		
**Diabetes (Yes)**		**51 (15)**	**11,1-19,0**		
**Dyslipidaemia (Yes)**		**94 (27,7)**	**22,8-32,6**		
**Anxiolytic/antidepressant medication (Yes)**		**170 (50,1)**	**44,7-55,6**		
**Age at menarche (years)**				**13,1 ±1,7**	**13**
**Age at menopause (years)**				**49,3 ±4,6**	**50**
**Age at first pregnancy (years)**				**25,1 ±5,4**	**24**
**Previous pregnancies (Yes)**		**286 (84,4)**	**80,3-88,4**		
**Abortions (Yes)**		**70 (24)**	**18,9-29,0**		
**Breastfeeding (Yes)**		**178 (52,5)**	**47,0-8,0**		
**Previous Tumour History**					
	**Previous benign breast disease**	**84 (24,8)**	**20,0-29,5**		
	**Previous malignant breast disease**	**14 (4,1)**	**1,9-6,4**		
	**Family history of breast cancer**	**109 (32,2)**	**27,0-37,3**		
	**Family history of ovarian cancer**	**20 (5,9)**	**3,2-8,5**		
	**Personal history of cancer**	**29 (8,6)**	**5,4-11,7**		

* CI: Confidence Interval. † SD: Standard Deviation

Table 2 shows the symptoms at diagnosis and the clinicopathological characteristics of the patients. In all, 44.8% had no symptoms at diagnosis, and 43.4% had a palpable tumour. Moreover, 41.9% were diagnosed through a screening programme, and a similar number (39.8%) were diagnosed by their primary care physicians. The most frequent histological type was Infiltrating Ductal Carcinoma (76.9%).

**Table 2:** Signs and symptoms at diagnosis, clinicopathological characteristics and stage. A Coruña, Spain, 2013-2015

		**n (%)**	95% CI*
**Symptoms at diagnosis**			
	**No clinical symptoms**	**152 (44,8)**	**39,4-50,3**
	**Palpable tumour in the breast or armpits**	**147 (43,4)**	**37,9-48,8**
	**Retraction of the nipple-areola complex**	**18 (5,3)**	**2,8-7,8**
	**Pain**	**15 (4,4)**	**2,1-6,8**
	**Swelling**	**7 (2,1)**	**0,4-3,7**
	**Discharge from the nipple**	**5 (1,5)**	**0,5-3,4**
	**Heat**	**4 (1,2)**	**0,3-3,0**
	**Ulcer in the skin of the breast**	**2 (0,6)**	**0,1-2,1**
**Palpable breast tumour according to surgeon**		**230 (67,8)**	**62,7-73,0**
**Palpable axillary adenopathy according to surgeon**		**61 (18)**	**13,8-22,2**
**Type of tumour**			
	**Infiltrating ductal carcinoma**	**260 (76,9)**	**72,3-81,6**
	**Infiltrating lobular carcinoma**	**27 (8)**	**4,9-11,0**
	**Ductal carcinoma in situ**	**26 (7,7)**	**4,7-10,7**
	**Mucinous carcinoma**	**6 (1,8)**	**0,2-3,3**
	**Invasive micropapillary carcinoma**	**4 (1,2)**	**0,3-3**
	**Apocrine carcinoma**	**3 (0,9)**	**0,2-2,6**
	**Mixed carcinoma**	**2 (0,6)**	**0,1-2,1**
	**Papillary carcinoma**	**2 (0,6)**	**0,1-2,1**
	**Metaplastic carcinoma**	**2 (0,6)**	**0,1-2,1**
	**Inflammatory carcinoma**	**2 (0,6)**	**0,1-2,1**
	**Infiltrating tubular carcinoma**	**2 (0,6)**	**0,1-2,1**
	**Paget’s disease of the breast**	**1 (0,3)**	**0,0-1,6**
**Nottingham Histologic Grading**			
	**Grade I**	**49 (14,7)**	**10,7-18,6**
	**Grade II**	**144 (43,1)**	**37,6-48,6**
	**Grade III**	**141 (42,)**	**36,8-47,7**
**Lymphatic vascular invasion (Yes)**		**67 (21,8)**	**17,0-26,6**
**Immunohistochemistry**			
	**Oestrogen receptor-positive**	**281 (82,9)**	**78,7-87,0**
	**Progesterone receptor-positive**	**248 (73,4)**	**68,5-78,2**
	**Her2-positive**	**45 (14,9)**	**10,8-19,1**
	**Ki67<20%**	**102 (38,2)**	**32,2-44,2**
	**Ki67≥20%**	**165 (61,8)**	**55,8-67,8**
**Molecular subtype**			
	**Luminal Her2-negative**	**236 (75,2)**	**70,2-80,1**
	**Triple-negative/basal-like**	**33 (10,5)**	**7,0-14,1**
	**Luminal B Her2-positive**	**24 (7,6)**	**4,5-10,7**
	**Non-luminal Her2**	**21 (6,7)**	**3,8-9,6**
**Stage at diagnosis**			
	**0**	**22 (6,5)**	**3,7-9,3**
	**I**	**149 (44,3)**	**38,9-49,8**
	**II**	**128 (38,1)**	**32,7-43,4**
	**III**	**35 (10,4)**	**7,0-13,8**
	**IV**	**2 (0,6)**	**0,1-2,1**

* CI: Confidence Interval

Table 3 describes the therapeutic management of the sample. In all, 78.5% of the women were offered surgery as the first treatment option; 49.7% had a lumpectomy, and 86.1% had sentinel node biopsy. Out of these patients, 10.0% were candidates for reconstructive surgery, of whom 88.2% underwent breast reconstruction with a tissue expander and subsequent replacement with a permanent prosthesis. Moreover, 53.4% ​​received chemotherapy, 84.1% received radiation therapy and 81.1% received hormone therapy.

**Table 3:** Therapeutic management of breast cancer. A Coruña, Spain, 2013-2015

		**n (%)**	95% CI*
**Adjuvant treatment (Yes)**		**266 (78,5)**	**73,9-83,0**
**Surgical Treatment**			
	**No Surgery**	**3 (0,9)**	**0,2-2,6**
	**Unilateral surgery**	**264 (77,9)**	**73,3-82,4**
	**Bilateral surgery**	**72 (21,2)**	**16,7-25,7**
**Type of surgery**			
	**Lumpectomy**	**167 (49,7)**	**44,2-55,2**
	**Oncoplastic surgery**	**81 (24,1)**	**19,4-28,8**
	**Modified radical mastectomy**	**40 (11,9)**	**8,3-15,5**
	**Simple mastectomy**	**34 (10,1)**	**6,7-13,5**
	**Skin-sparing mastectomy**	**9 (2,7)**	**0,8-4,5**
	**Skin and nipple-sparing mastectomy**	**3 (0,9)**	**0,2-2,6**
	**Axillary lymphadenectomy only**	**1 (0,3)**	**0,0-1,6**
	**Supraclavicular mass resection**	**1 (0,3)**	**0,0-1,6**
**Areola-Nipple Complex Resection (Yes)**		**92 (27,1)**	**22,3-32,0**
**Surgical margins affected (Yes)**		**48 (15,0)**	**10,9-19,1**
**Sentinel Lymph Node Biopsy (Yes)**		**292 (86,1)**	**82,3-90,0**
**Positive Sentinel Lymph Node (Yes)**		**131 (44,9)**	**39,0-50,7**
**Extranodal extension**			
	**Yes**	**77 (23,0)**	**18,3-27,6**
	**Indeterminate**	**88 (26,3)**	**21,4-31,1**
**Axillary lymphadenectomy (Yes)**		**91 (26,8)**	**22,2-32,0**
**Lymph node status in Lymphadenectomy**			
	**Micrometastasis**	**7 (9,9)**	**2,2-17,5**
	**Macrometastasis**	**64 (90,1)**	**82,5-97,8**
**Breast Reconstruction (Yes)**		**34 (10,0)**	**6,7-13,4**
	**Immediately**	**27 (79,4)**	**4,9-11,0**
	**Deferred**	**7 (20,6)**	**0,4-3,7**
**Type of reconstruction**			
	**Tissue expander**	**30 (88,2)**	**72,5-96,7**
	**Prosthesis**	**4 (11,8)**	**3,3-27,4**
**Chemotherapy (Yes)**		**181 (53,4)**	**47,9-58,8**
**Radiotherapy (Yes)**		**285 (84,1)**	**80,0-88,1**
**Hormone therapy (Yes)**		**275 (81,1)**	**76,8-85,4**
**Biological therapy anti-Her2 (Yes)**		**40 (11,8)**	**8,2-15,4**
**Rehabilitation of lymphedema (Yes)**		**55 (16,2)**	**12,1-20,3**
**Oncopsychology (Yes)**		**43 (12,7)**	**9,0-16,4**

* CI: Confidence Interval

The results of the quality of life questionnaires (European Organisation for Research and Treatment of Cancer (EORTC) QLQ C-30 and Br23) are shown in Table 4. At the time of diagnosis, the highest quality of life scores were those for physical function, role and body image, with scores higher than 90%. The lowest scores were for future prospects and sexual enjoyment, with values ​​lower than 60%. After the treatments were complete, the dimensions that were negatively and significantly modified were physical function, role, body image, financial concerns and symptoms such as fatigue, pain, dyspnoea and those related to the breast, arm and adverse effects of systemic therapies. Scores that were significantly improved after treatment were emotional function and future prospects. The Global Health Status also improved after treatment, but the improvement was not statistically significant.

**Table 4:** Baseline measurements and post-treatment quality of life with the EORTC QLQ-30 and Br-23 questionnaires and the Anxiety as State and as Trait with the STAI questionnaire. A Coruña, Spain, 2013-2015

		**Baseline**			**Post-treatment**		**Difference**	**p**
		**n**	Mean±SD*		**n**	Mean±SD*		
**EORTC QLQ C-30**								
**Global Health Status**		**181**	**69,2 ±21,1**		**181**	**72,0 ±21,6**	**2,7 ±23,4**	**0,167**
**Functional scales**								
	**Physical function**	**185**	**92,3 ±12,4**		**185**	**84,6 ±16,3**	**-7,7 ±15,1**	**<0,001**
	**Role function**	**182**	**93,3 ±14,3**		**182**	**85,4 ±22,2**	**-7,9 ±23,3**	**<0,001**
	**Emotional function**	**183**	**63,0 ±25,0**		**183**	**74,4 ±23,7**	**11,3 ±25,2**	**<0,001**
	**Cognitive Function**	**183**	**84,5 ±18,2**		**183**	**85,0 ±20,8**	**0,4 ±23,3**	**0,535**
	**Social function**	**182**	**88,4 ±18,4**		**182**	**86,3 ±22,1**	**-2,1 ±22,8**	**0,299**
**Scales of symptoms/items**								
	**Fatigue**	**186**	**15,8 ±17,5**		**186**	**26,4 ±23,0**	**-10,6 ±19,9**	**<0,001**
	**Nausea andvomiting**	**186**	**4,5 ±13,8**		**186**	**5,7 ±13,9**	**-1,2 ±17,5**	**0,134**
	**Pain**	**186**	**10,1 ±15,3**		**186**	**19,7 ±23,0**	**-9,6 ±21,6**	**<0,001**
	**Dyspnoea**	**182**	**10,3 ±19,0**		**182**	**14,6 ±23,9**	**-4,4 ±26,3**	**0,034**
	**Insomnia**	**184**	**31,7 ±29,8**		**184**	**29,3 ±29,1**	**2,3 ±32,5**	**0,384**
	**Loss of appetite**	**185**	**14,0 ±21,6**		**185**	**12,2 ±22,4**	**1,8 ±26,4**	**0,408**
	**Constipation**	**182**	**12,4 ±23,0**		**182**	**15,6 ±23,7**	**-3,1 ±27,5**	**0,153**
	**Diarrhoea**	**182**	**5,7 ±16,4**		**182**	**7,3 ±16,3**	**-1,6 ±20,5**	**0,248**
	**Financial Concerns**	**181**	**5,0 ±16,7**		**181**	**10,7 ±24,5**	**-5,7 ±22,7**	**0,001**
**EORTC QLQ Br-23 Breast Cancer Module**								
**Functional scales**								
	**Body image**	**180**	**94,2 ±13,2**		**180**	**85,1 ±23,0**	**-9,15 ±22,9**	**<0,001**
	**Sexual function**	**153**	**79,4 ±23,7**		**153**	**80,5 ±21,8**	**1,09 ±21,4**	**0,454**
	**Sexual enjoyment**	**58**	**55,7 ±32,7**		**58**	**55,2 ±30,3**	**-0,57 ±22,1**	**0,841**
	**Future perspectives**	**179**	**46,0 ±33,5**		**179**	**54,6 ±33,1**	**8,57 ±37,9**	**0,005**
**Scales of symptoms/items**								
	**Adverse effects of systemic therapies**	**184**	**14,4 ±13,4**		**184**	**22,7 ±19,6**	**-8,36 ±18,9**	**<0,001**
	**Breast symptoms**	**181**	**12,1 ±14,8**		**181**	**23,0 ±19,9**	**-10,97 ±20,8**	**<0,001**
	**Arm symptoms**	**181**	**9,2 ±14,5**		**181**	**16,4 ±18,3**	**-7,18 ±17,9**	**<0,001**
	**Worry about hair loss**	**29**	**26,4 ±37,1**		**29**	**26,4 ±36,0**	**0 ±46,3**	**0,972**
**State anxiety**		**169**	**29,7 ±13,9**		**169**	**19,4 ±11,8**	**10,3 ±14,7**	**<0,001**
**Trait anxiety**		**165**	**22,1 ±10,5**		**165**	**20,0 ±10,2**	**2,0 ±9,8**	**0,009**
			**n (%)**			**n (%)**		
**Level of state anxiety**		**169**			**169**			**<0,001**
	**Light**		**27 (16,0)**			**67 (39,6)**		
	**Moderate**		**66 (39,1)**			**70 (41,4)**		
	**Severe**		**76 (45,0)**			**32 (18,9)**		
**Level of trait anxiety**		**165**			**165**			**0,470**
	**Light**		**59 (35,8)**			**68 (41,2)**		
	**Moderate**		**81 (49,1)**			**74 (44,8)**		
	**Severe**		**25 (15,2)**			**23 (13,9)**		

* SD: Standard Deviation.

After the multivariate logistic regression analysis using the median of the Global Health Status domain and age, level of education, Charlson score, anxiolytic medication, previous pregnancies, family history of cancer and the retraction of the areola-nipple complex as covariables, we found that the independent variables that predicted values ​​lower than the median in the Global Health Status were education level, Charlson score, anxiolytic medication, previous pregnancies and retraction of the nipple. The higher educational level (low vs high) was associated with improved quality of life (OR=0.48), while the presence of comorbidities (OR=2.07), the use of anxiolytic drugs=1.61), previous pregnancies (OR=1.99) and nipple retraction (OR=4.50) were found to increase the risk of a poorer quality of life (Table 5).

**Table 5:** Logistic regression models were used to predict the variables associated with the lowest Global Health Status score (EORTC QLQ C-30 questionnaire) and severe levels of anxiety as a state and as a trait (STAI questionnaire) at baseline. A Coruña, Spain, 2013-2015

Variables		**Global Health Status**	**State Anxiety**	**Trait Anxiety**
		OR* (CI† 95%)	OR* (CI† 95%)	OR* (CI† 95%)
**Age (years)**		**0,98 (0,96-1,01)**	**0,99 (0,97-1,01)**	**0,98 (0,94-1,02)**
**Education level**				
	**Low**	**1**		
	**Low vs middle**	**0,98 (0,51-1,89)**		
	**Low vs high**	**0,48 (0,25-0,94)**		
**Civil status**				
	**Single**		**1**	
	**Single vs married/partner**		**2,28 (1,07-4,88)**	
	**Single vs divorced**		**1,50 (0,48-4,67)**	
	**Single vs widowed**		**1,52 (0,57-4,08)**	
**Employment situation**				
	**Active**			**1**
	**Active vs inactive**			**3,79 (1,04-13,82)**
	**Active vs housewife**			**4,12 (1,37-12,38)**
	**Active vs retired**			**3,07 (0,91-10,34)**
**Housework**				
	**No help**			**1**
	**No help vs shared tasks**			**1,22 (0,57-2,61)**
	**No help vs other situation**			**1,21 (0,42-3,47)**
**Charlson Comorbidity Index**		**2,07 (1,29-3,30)**		
**Anxiolytic/antidepressant medication**		**1,61 (1,01-2,56)**	**2,13 (1,35-3,38)**	
**Previous pregnancies**		**1,99 (1,01-3,89)**		
**Age at first pregnancy**				**0,97 (0,90-1,04)**
**Family history of breast cancer**		**0,71 (0,43-1,17)**		**0,49 (0,22-1,10)**
**Nipple retraction**		**4,5 (1,21-16,73)**	**1,96 (0,67-5,71)**	**2,32 (0,60-9,05)**
**Breast swelling**			**6,35 (0,70-57,84)**	**5,32 (1,03-27,42)**
**Stage at diagnosis (OI-II vs III-IV)**			**1,55 (0,74-3,28)**	**2,60 (1,05-6,47)**
*Cox and Snell’s R2*		**0,11**	**0,075**	**0,088**

* OR: Odds Ratio. † CI: Confidence Interval

Table 4 shows the scores for anxiety as a state and as a trait at diagnosis and at the end of treatment. At the time of diagnosis, 45% of the women had severe levels of state anxiety, while 15.2% had severe levels of trait anxiety. After treatment, only 18.9% had severe levels of state anxiety, which demonstrates a statistically significant improvement (p<0.001). The same trend was observed for trait anxiety (p=0.009).

After the multivariate logistic regression analysis was performed to determine the variables associated with severe levels of anxiety as a state, it was found that the independent variables of marital status and anxiolytic medication predicted severe levels of state anxiety. Women who were married or who had a partner exhibited a 2.28-fold higher risk of severe anxiety compared with single women, and those who took anxiolytic medication were 2.13 times more likely to have severe anxiety (Table 5). 

Regarding trait anxiety, the independent variables that affect the levels of severe trait anxiety were employment situation, breast swelling and stage at diagnosis. Women who did not work or who were housewives exhibited higher levels of trait anxiety than those who worked (OR=3.79 and 4.12, respectively). Women who presented with breast swelling as a symptom at diagnosis were 5.32 times more likely to experience severe levels of trait anxiety compared with women with less invasive disease stages (0, I, II) at the time of diagnosis; women with more advanced disease stages (III, IV) exhibited a similar trend (OR=2.60) (Table 5).

## Discussion

This study reveals that the health problems most commonly identified at the time of diagnosis involve the psychological field, as they affect the dimensions that are related to emotional functionality, future perspectives, insomnia, anxiety and sexual enjoyment. These results are in agreement with the results of similar studies^15^ ^-^ ^16^ and with the EORTC reference values ​​for the questionnaires used^17^. The Global Health Status (GHS), which is considered the best reflection of the subjective perception of well-being and quality of life in the QLQ C-30 questionnaire, was, at the time of diagnosis, similar to that described in other studies^16^ ^,^ ^18^ ^-^ ^19^. 

Quality of life is initially negatively affected by previous comorbidities and anxiolytic medication, with the understanding that these women have a poorer baseline quality of life due to their associated previous pathologies^20^ ^-^ ^21^. Nipple retraction is also associated with lower quality of life scores. This sign of disease is usually indicative of more advanced stages of breast cancer, namely, a stage in which the disease usually manifests itself in a more systemic way that affects the overall quality of life to a greater extent.

The education level also influences the quality of life. Women with a university education have higher quality of life scores, which can be derived from a higher cultural level and knowledge, as well as better-paying jobs. This provides them with access to information and allows them to have a greater number of tools, resources and coping strategies to adapt to the disease. Likewise, more economic resources give them solvency to meet the needs generated by this new health situation^22^ ^-^ ^23^.

No significant differences were found between various stages at diagnosis and quality of life. This is consistent with the findings of previous studies, suggesting that, despite the excellent prognosis for stage 0 women, the diagnosis of breast cancer is stressful and may result in a pattern of psychological morbidity similar to that experienced by women with invasive disease^22^ ^,^ ^24^.

Although different studies have reported a significant improvement in the Global Health Status (GHS)^16^ ^,^ ^25^, in our study, the improvement between the baseline and post-treatment scores did not reach statistical significance. Based on the literature review, it is observed that the quality of life improves as the follow-up periods are extended^12^ ^,^ ^26^. In one study on the impact and time of deterioration of the quality of life in patients with breast cancer^19^, the GHS at three months had worsened with respect to the baseline measurement. The evidence provided by the results of a prospective investigation after five years of follow-up after breast surgery^27^ states that most positive changes in quality of life occur between one and two years after treatments. In a study that measured the long-term quality of life in breast cancer survivors^18^, it was observed that five years after diagnosis, the GHS is not affected by the severity of the disease and/or the treatments received, which indicates that the quality of life at five years seems to be affected by the same factors that affect the general population (generally age and comorbidities)^20^. 

In this study, after treatment, the general symptomatology of the patients and the physical and role functions significantly worsened. This symptomatology tends to increase in the months after surgery due to the treatments received such as chemotherapy, radiotherapy and hormonal therapies. These findings are consistent with those of previous studies^28^ ^-^ ^29^. A study on the changes that occur in the quality of life of patients with breast cancer^30^ showed that after six months, body image scores and adverse effects of systemic therapies worsened. In that study, the future prospects dimension improved, which is what occurred in this cohort, as did the symptoms related to the breast and the arm. Thus, most aspects of quality of life, including physical function and residual effects of adjuvant treatments, will be recovered in longer follow-up periods for most patients^31^.

This cohort demonstrated a significant worsening of body image, and although the difference was not statistically significant, this cohort also demonstrated a decrease in sexual function and enjoyment, which is consistent with the results of other investigations^21^ ^-^ ^22^ ^,^ ^32^. Previous breast size, type of surgery and outcome (asymmetry, changes in skin integrity) may pose a threat to the breast. Changes in these factors can significantly affect the body image that each woman has of herself^33^. Over the years, the ways in which the breasts have been portrayed have changed, but the message remains essentially the same. Female breasts are considered symbols of intrinsic femininity, sexual desire and maternal comfort and relief. Whether the breasts are presented in a subtle and evocative way or explicitly displayed, they are central to the opinions of what many people consider “being a woman.” It is therefore understandable that any real or potential threat to a woman’s breasts is stressful. Given the importance society places on female breasts, it is not surprising that both surgical treatment and the disease itself have a devastating impact on women’s confidence and self-esteem, as they may feel less attractive than before and have decreased body image scores, which negatively affect their sexuality.

Between baseline and post-treatment measurements, the emotional function scale and the future prospects of the participants have improved, as in previous studies^30^ ^,^ ^32^. Once the initial shock, which may arise when a patient receives a diagnosis of breast cancer, is overcome, the process of healing is initiated, which increases a woman’s confidence in the possibility of a cure. This in turn decreases the concern for the future and the onset of the disease. Thus, we observed that, even though the improvement was not significant, insomnia improves with respect to the initial measurement. Women are relieved and seem to have a more positive future outlook once adjuvant treatments have been completed, but this improvement can also be attributed to the patient’s ability to adapt to new situations^27^ or the coping strategies that the women have applied^34^ ^-^ ^35^. In a clinical trial that compared two first-line chemotherapy treatment options for advanced breast cancer^36^, it is notable that improvement in emotional function, in addition to its association with decreased pain and other symptomatology, may simply reflect the fact that some action has been undertaken, regardless of what that action is, which may be an indication of hope in a life-threatening situation.

The levels of anxiety that were found show a prevalence of psychomorbidity similar to that in previous studies^37^ ^-^ ^38^, which have reported a prevalence of anxiety and psychological distress of approximately 20-30% among cancer patients. This anxiety is related to the fear of death and the uncertainty about the future, the disease and the treatment course^37^. Severe anxiety, the diagnosis of cancer and the treatments received impact the patient’s quality of life and may cause deterioration of the quality of life as well as the way in which he or she adapts to his or her new situation^39^. The multivariate analysis revealed that the variables of marital status and the use of anxiolytic medication could predict severe levels of state anxiety in patients with breast cancer. 

Anxiety is highest in women who are treated with anxiolytic or antidepressant medications. This is consistent with other studies that have shown that women with a psychological disturbance who take anxiolytic or antidepressant medications present greater psychological somatization and phobic anxiety and are more susceptible to severe levels of anxiety and/or depression^5^ ^-^ ^6^. Obviously, the collection of descriptive information at baseline does not allow a causal inference to be made, but rather, it simply allows us to describe the association between the variables studied.

On the contrary, more severe anxiety levels have been observed among married or partnered women. It is possible that, for many of the women diagnosed with breast cancer, the concern for their families is greater than the concern they have for themselves and that this concern becomes a stress-generating variable for them, which is consistent with the findings of previous studies^40^ ^-^ ^41^. This circumstance can also be related to feelings of insecurity about the acceptance of the disease by their partners, with the added fear that their partners may end the relationship because of the disease or leave them for another woman^40^ ^-^ ^42^.

Anxiety as a trait is the tendency to perceive situations as threatening, which then raises the level of anxiety. This type of anxiety is strongly influenced by the patient’s work situation, the stage at diagnosis and breast swelling as an initial symptom attributable to breast cancer.

Women who do not work, either because they are housewives, are unemployed or have time off, have poorer scores of trait anxiety. This increased anxiety in women without paid employment may be due to concerns about income and financial distress^40^ ^,^ ^42^ ^-^ ^43^. They also have more free time and thus more time to reflect on the disease, its treatments and its onset. The lack of employment also leads to a reduction in social relationships. Contrary to what was found in this study, a study in Korea that investigated the association between socioeconomic status and altered body image and quality of life among breast cancer patients^23^ found that working women are more anxious than retired women or housewives. This may be due to the pressure to which they are subjected with respect to maintaining their body image in the workplace and also to the difficulties they find in dressing appropriately for work. They feel that they are easily identified by their alopecia and other changes in appearance, which can hamper the social activities and work performance of women with breast cancer.

The timing of the evaluation of psychological reactions can be decisive, as stress-coping responses may change over time after the initial diagnosis^37^. This suggests that women may gradually accept their illness and feel less anxious. Sometimes, excess optimism is observed as a form of denial through which they attempt to minimize the severity of their condition.

The levels of state anxiety and trait anxiety have been shown to improve significantly after the completion of treatments, which is consistent with what is reported in the literature^16^ ^,^ ^44^. In a study on anxiety, depression and quality of life in Malaysia^16^, the anxiety levels were reduced at six and twelve months compared with baseline. Despite significant improvement in anxiety levels, several investigators have reported that women with breast cancer continue to have serious concerns about their disease several years after diagnosis and surgery and that they suffer from long-term psychological stress and depression^(37, 45)^. 

Elevated anxiety intensifies physical symptoms and influences the overall quality of life. A gradual decrease in psychological stress can result in an improvement in the quality of life subscales, such as body image and emotional function^46^. Elevated anxiety levels at baseline may be associated with a lack of information on breast cancer as well as a poor understanding of the course of the disease and the effects of treatments. As patients acquire knowledge, the anxiety they experience about the unknown decreases. The literature shows that adherence to treatment decreases anxiety^47^.

The limitations of this study are as follows:

Selection bias: to reduce this bias, all patients with a positive biopsy for breast cancer were consecutively selected during the study period. The consistency of the results compared with similar studies provides external validity.

Bias of information: validated questionnaires with trained interviewers have been used to minimize this bias.

Confounding bias: a multivariate analysis using logistic regression techniques was used to control for the confounding effects of the different variables and the presence of third variables, such as sociodemographic and comorbidity variables.

This study provides knowledge about the characteristics of breast cancer and the comorbidities of the patients, as well as information about their quality of life and anxiety at the time of diagnosis and after the end of treatment. The identification of these factors will allow us to begin initiatives and interventions that will improve the quality of life and the ability to adequately manage the anxiety of these patients. The need for a multidisciplinary approach for these patients is evidenced by the different dimensions that affect the quality of life; different health professionals could then collaborate on the improvement of quality of life. The determination of the factors that predict changes in quality of life provides important information for clinical practice and should be used for the development of evidence-based guidelines in the design of breast cancer follow-up protocols. Nurses and other health professionals who are involved in the care of breast cancer patients should assess the expectations and needs of individual survivors and focus the care accordingly, because quality of life and anxiety are, to a large extent, individual and subjective perceptions.

## Conclusions

Quality of life is decreased in women with a low level of education, those with other comorbidities, those who take anxiolytic medication, those with previous pregnancies and in women with nipple retraction. The quality of life measured before and after treatment changed in a positive and significant way in the following dimensions: emotional function and future prospects. In turn, negative changes occurred in the following dimensions: physical function, role function, fatigue, pain, dyspnoea, financial concerns, body image, symptoms of systemic therapies and symptoms associated with the breast and arm.

Anxiety is increased in married women, women who do not work, those who take anxiolytic medication and in those with breast swelling and advanced-stage disease. Anxiety as a state and as a trait is decreased significantly between pre- and post-treatment.

This study determined the most frequent health problems in women with breast cancer in our field. The need to reinforce care, support and information in dimensions such as emotional function, sexual enjoyment and body image is emphasized. In turn, the support of initiatives that are already established and that foster and guide the development of future interventions, is vital.
